# Vitamin D receptor signaling in inflammatory senescence–associated skin aging: Mechanisms and therapeutic potentials

**DOI:** 10.1002/ccs3.70088

**Published:** 2026-05-27

**Authors:** Liancheng Guan, Fan Yang, Meijuan Li, Yujia Chen, Zexin Zhao, Hongxia Li, Deping Luo, Qian Li, Yunzhi Chen

**Affiliations:** ^1^ The Second Affiliated Hospital of Guizhou University of Traditional Chinese Medicine Guiyang Guizhou China; ^2^ Guizhou University of Traditional Chinese Medicine Guiyang Guizhou China

**Keywords:** cellular senescence, immunoregulation, inflammation, signaling, VDR, vitamin D

## Abstract

Vitamin D receptor (VDR) signaling plays a crucial role in skin homeostasis and represents a promising therapeutic target for inflammatory senescence–associated skin aging. This review examines the roles of VDR signaling in inflammatory senescence‐associated skin aging. We summarize the actions of VDR through chromatin modification, transcriptional regulation, and coregulator recruitment, which collectively modulate inflammation and cellular senescence in aging skin. Recent findings highlight the protective role of VDR against inflammatory responses and aging processes through multiple pathways, including NF‐κB suppression and regulation of senescence‐associated pathways. The review discusses cell‐type specific VDR regulation in keratinocytes, immune cells, dermal fibroblasts, and stem cells, emphasizing their distinct roles in skin homeostasis. Current therapeutic approaches encompass vitamin D–based treatments, traditional medicine–derived bioactive compounds, and novel drug delivery systems. Research challenges include limitations in monitoring VDR activity in aging skin and developing effective tissue‐specific delivery methods. This analysis emphasizes the therapeutic potential of VDR modulation in aging‐related skin conditions and highlights priorities for future investigation of VDR‐mediated regulation in inflammatory senescence–associated skin diseases.

## Graphical abstract

Vitamin D receptor (VDR) signaling is implicated in inflammatory senescence‐associated skin aging, a growing health concern in aging populations where cellular senescence and chronic inflammation converge to create complex pathophysiological conditions. VDR, a nuclear receptor that responds to active vitamin D3, is widely found in skin cells including keratinocytes, fibroblasts, immune cells, and stem cells, where it regulates gene expression through chromatin modification, coregulator recruitment, and transcriptional control. The aging process significantly impairs the vitamin D/VDR system by reducing vitamin D synthesis capacity and decreasing VDR expression and epigenetic changes, which contribute to compromised skin homeostasis, barrier dysfunction, and delayed wound healing. Cellular senescence in aging skin triggers the senescence‐associated secretory phenotype (SASP), releasing pro‐inflammatory cytokines (IL‐6 and TNF‐α) and matrix metalloproteinases that promote chronic inflammation and tissue degradation. VDR signaling counteracts these aging processes by suppressing NF‐κB pathway activation, modulating MAPK and PI3K/Akt signaling, and interacting with p53 and p63 pathways to regulate DNA repair, cellular differentiation, and senescence. Current therapeutic applications include topical vitamin D analogs like calcipotriol for psoriasis, while emerging approaches involve traditional Chinese medicine compounds (such as berberine) that modulate VDR signaling, as well as novel drug delivery systems including nanoformulations and liposomal carriers. Future research priorities include developing improved methods for studying VDR signaling in human skin using single‐cell technologies, creating personalized treatments based on VDR genetic polymorphisms and designing tissue‐specific delivery systems that effectively target senescent cells while minimizing systemic effects.

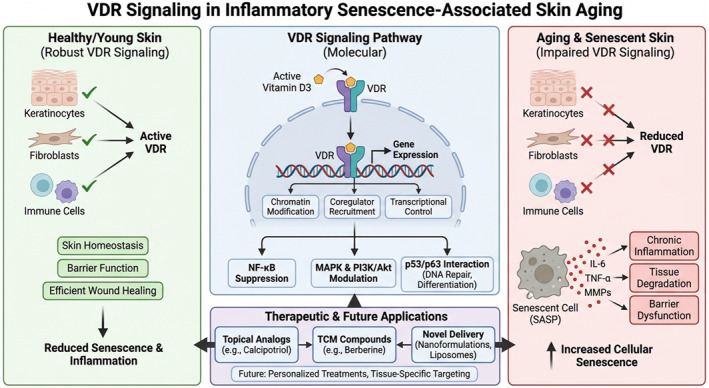

## 1 INTRODUCTION

Inflammatory senescence–associated skin diseases represent a significant and growing health concern, particularly in aging populations.^1^ These conditions are characterized by the convergence of two critical biological processes: cellular senescence and chronic inflammation, which together create a complex pathophysiological environment in the skin.^2^ With the global demographic shift toward an aging population, these disorders are becoming increasingly prevalent, impacting both quality of life and healthcare resources.^3^ The skin, as the body's largest organ and the primary barrier against environmental stressors, is particularly susceptible to senescence‐associated changes, which can manifest as various inflammatory conditions, including psoriasis, atopic dermatitis, and other chronic inflammatory skin disorders.^4^ Understanding these diseases is crucial as they not only affect physical health but also have significant psychological and social implications for patients.^5^

The vitamin D receptor (VDR) is a crucial nuclear receptor that plays vital roles in various physiological functions in skin health.^6^ First identified and cloned in 1988 by Andrew Baker and colleagues, VDR belongs to the nuclear receptor 1 superfamily and functions as a key sensor for the active form of vitamin D3 (calcitriol or 1α,25‐dihydroxy vitamin D3).^7^ Upon activation, VDR triggers diverse specific gene expressions essential for skin development and metabolism. The receptor can be found in various cellular locations including the nucleus, cytosol, and plasma membrane, though it is predominantly localized in the nucleus.^8^ Remarkably, VDR is highly conserved among various organisms, from mammals to amphibians, with even the worm *Caenorhabditis elegans* possessing a related homolog called DAF‐12.^9^, ^10^ This evolutionary conservation underscores the fundamental importance of VDR in biological systems.

Furthermore, VDR expression is widely distributed across numerous skin cell types and demonstrates remarkable tissue specificity in its function.^11^, ^12^ Its activity is carefully regulated through autoregulation by its native agonist, both in vivo and at the cellular level.^13^ The importance of VDR signaling in skin health is underscored by its involvement in multiple noncommunicable diseases, including various skin conditions related to inflammaging and immunosenescence.^14^ Through its interaction with retinoid X receptor heterodimers and subsequent binding to vitamin D response elements on DNA, VDR regulates genes crucial for cellular homeostasis, immune function, and skin barrier maintenance.^15^ Current evidence regarding the use of vitamin D and its synthetic analogs in treating noncommunicable skin diseases continues to show promise, though more comprehensive clinical validation is still needed.^16^, ^17^ This emerging therapeutic potential could be further enhanced through complementary approaches, particularly involving traditional medicines and bioactive compounds that target vitamin D signaling pathways.^18^ Such integrative strategies may offer new opportunities for developing more effective and comprehensive therapies for age‐related dermatological conditions.

In this review, we examine the fundamental roles of vitamin D and VDR signaling in skin health, focusing on inflammatory senescence‐associated skin diseases. We explore the intricate relationship between cellular senescence and inflammation in aging skin and detail cell‐type specific VDR regulation in keratinocytes, immune cells, dermal fibroblasts, and stem cells. We investigate the molecular mechanisms of VDR action and its impact on skin homeostasis through barrier function, inflammatory responses, and senescence control. Additionally, we discuss the modulation of VDR signaling by bioactive compounds and various therapeutic approaches. Finally, we address current challenges and future directions in developing effective treatments for inflammatory senescence‐associated skin diseases.

## 2 VITAMIN D/VDR BIOLOGY AND ITS IMPLICATION IN SKIN HOMEOSTASIS

### 2.1 VDR structure and expression in skin cells

The VDR gene, encoding the VDR protein, is strategically located on chromosome 12q13.11 in humans and chromosome 15 in mice.^19^ The VDR gene comprises nine exons and exhibits multiple isoforms, allowing for diverse functional variations.^20^, ^21^ Several key polymorphic sites have been identified within the VDR gene, including VDR ApaI, BsmI, TaqI, and FokI. These genetic variations significantly influence mRNA expression patterns, protein translation efficiency, and ultimately, VDR transcriptional activity.^8^, ^22^, ^23^ In skin tissue, VDR is abundantly expressed across various cell types, including keratinocytes, melanocytes, fibroblasts, and immune cells.^24^, ^25^, ^26^ For instance, in keratinocytes, VDR expression is particularly high and plays a crucial role in regulating cell differentiation and proliferation. The regulation of VDR expression in skin cells is complex and dynamic, influenced by multiple factors including the presence of its natural agonist 1α,25‐dihydroxyvitamin D3, cellular molecules such as parathyroid hormone and retinoic acid, and tissue‐specific regulatory elements.^13^, ^27^ This intricate regulation ensures appropriate VDR signaling in skin tissue, which is essential for maintaining skin barrier function, immune response, and overall skin health.

### 2.2 Molecular mechanism of VDR signaling

**FIGURE 1:**
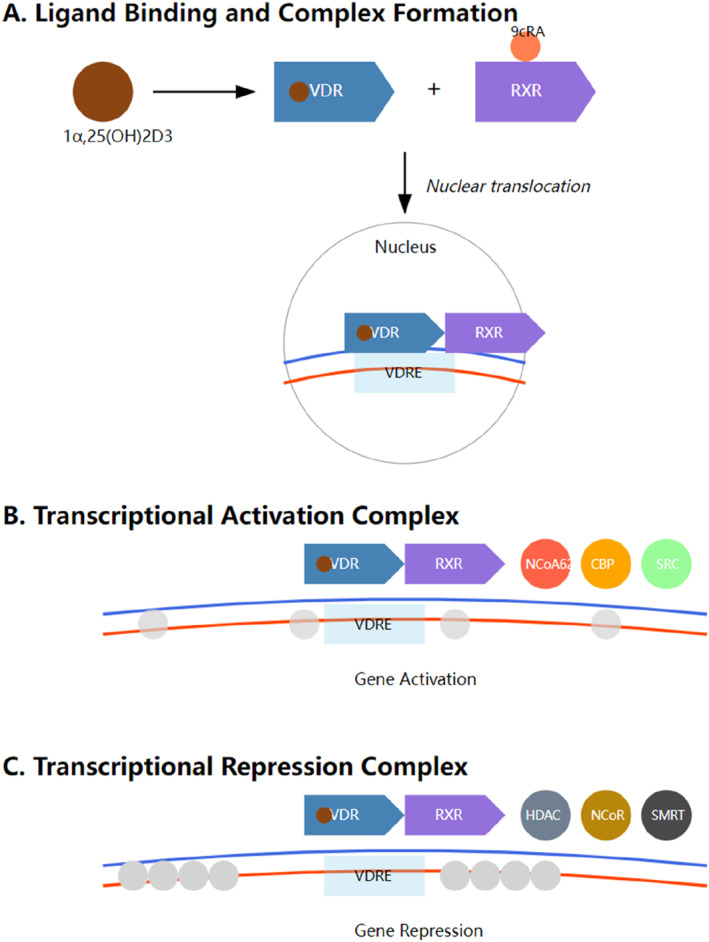
Molecular mechanisms of VDR‐mediated transcriptional regulation. (A) Ligand binding and complex formation: The active vitamin D metabolite (1α,25(OH)2D3) binds to vitamin D receptor, which then forms a heterodimer with RXR. This complex undergoes nuclear translocation and binds to vitamin D response elements (VDRE) in target gene promoters. (B) Transcriptional activation complex: Upon binding to VDRE, the VDR–RXR heterodimer recruits coactivator proteins including NCoA62 (nuclear receptor coactivator), CREB‐binding protein, and steroid receptor coactivator (SRC) (SRC), leading to gene activation. (C) Transcriptional repression complex: Alternatively, the VDR–RXR heterodimer can recruit corepressor proteins including HDAC (histone deacetylase), NCoR (nuclear receptor corepressor), and SMRT (silencing mediator for retinoid and thyroid hormone receptors), resulting in gene repression.

The molecular activation of VDR involves a complex cascade of events triggered by ligand binding. When the active form of vitamin D (1α,25‐dihydroxy vitamin D3) binds to VDR, it initiates a conformational change that facilitates the formation of a heterodimer with the retinoid X receptor (RXR).^28^ This VDR‐RXR complex then translocates to the nucleus, where it interacts with specific vitamin D response elements in target gene promoters.^29^ The transcriptional activity of VDR is tightly regulated through interactions with various coactivators and corepressors. In the presence of ligands, the complex recruits coactivators such as steroid receptor coactivators, CREB‐binding protein (CBP), and nuclear receptor coactivator 62 kDa (NCoA62), leading to chromatin remodeling and transcriptional activation.^30^, ^31^, ^32^, ^33^, ^34^, ^35^, ^36^ Conversely, in the absence of ligands, VDR associates with corepressors like nuclear receptor corepressor (NCoR), histone deacetylases (HDACs), and silencing mediator for retinoid and thyroid hormone receptors (SMRT), resulting in transcriptional repression^37^, ^38^, ^39^, ^40^ (Figure 1).

The structural organization of VDR is critical for its function as a transcription factor. The receptor consists of several distinct domains, including the N‐terminal A/B region, the highly conserved DNA‐binding C domain containing two zinc finger motifs, the flexible hinge D domain, and the ligand‐binding E domain.^41^, ^42^, ^43^ The DNA‐binding domain (DBD) specifically recognizes vitamin D response elements through its zinc finger motifs, while the ligand‐binding domain forms a hydrophobic pocket that accommodates 1α,25‐dihydroxy vitamin D3 and mediates interactions with coregulatory proteins.^44^, ^45^, ^46^ Posttranslational modifications, including phosphorylation, SUMOylation, and ubiquitination, provide additional layers of regulation by modulating VDR's stability, localization, and transcriptional activity.^47^, ^48^, ^49^ These modifications can either enhance or suppress VDR function, demonstrating the complex nature of VDR regulation in cellular environments.

### 2.3 Vitamin D metabolism in skin and the role of VDR signaling in skin homeostasis

The skin serves as the primary site for vitamin D synthesis and metabolism, playing a crucial role in maintaining both local and systemic vitamin D homeostasis.^50^ When the skin is exposed to UVB radiation, 7‐dehydrocholesterol in keratinocytes is converted to pre‐vitamin D3, which then undergoes thermal isomerization to form vitamin D3.^50^, ^51^ This vitamin D3 is subsequently metabolized to 25‐hydroxyvitamin D3 by CYP2R1 in the liver and further converted to the active form, 1α,25‐dihydroxyvitamin D3, by CYP27B1 in the kidneys and local tissues including the skin. Notably, skin cells express all the enzymatic machinery necessary for vitamin D metabolism, including CYP27B1 and CYP24A1, allowing for local production and regulation of active vitamin D.^52^, ^53^ The VDR signaling pathway in the skin is essential for maintaining epidermal barrier function, keratinocyte differentiation, hair follicle cycling, and immune response regulation.^54^ In keratinocytes, VDR activation by vitamin D promotes the expression of genes involved in differentiation (such as involucrin and loricrin), barrier formation (including filaggrin and ceramide synthesis enzymes), and antimicrobial defense (like cathelicidin and defensins).^55^, ^56^ The importance of VDR in skin homeostasis is evidenced by studies of VDR knockout mice and humans with VDR mutations, which exhibit defective epidermal barrier function, alopecia, and increased susceptibility to skin infections and inflammation.^57^ Additionally, VDR signaling regulates the skin immune system by modulating the function of various immune cells, including T cells, dendritic cells, and Langerhans cells, thereby contributing to both innate and adaptive immune responses in the skin.^58^ The complex interplay between vitamin D metabolism and VDR signaling in skin cells is further modulated by factors such as age, UV exposure, inflammation, and various environmental stressors, highlighting the dynamic nature of this system in maintaining skin health.^59^

### 2.4 Age‐related changes in the vitamin D/VDR system

The aging process significantly impacts the vitamin D/VDR system through multiple mechanisms, leading to decreased vitamin D synthesis, metabolism, and signaling efficiency.^60^ With increasing age, the skin's capacity to produce vitamin D decreases markedly due to reduced levels of 7‐dehydrocholesterol and diminished UV exposure response.^61^ An early study found that aging significantly reduces the capacity of the human skin to produce vitamin D3, as decreased epidermal 7‐dehydrocholesterol concentrations in elderly individuals substantially impair cutaneous vitamin D photosynthesis compared to younger adults.^61^ Age‐associated decline in VDR expression has been observed across various tissues, particularly in the skin, bone, and immune cells, contributing to reduced vitamin D responsiveness.^62^ This decrease in VDR expression could lead to impaired nuclear translocation of the receptor and altered coregulator recruitment patterns. Furthermore, epigenetic changes occurring with age, such as DNA methylation and histone modifications in the VDR gene promoter region, can lead to suppressed VDR expression.^63^, ^64^ The age‐related deterioration of the vitamin D/VDR system has been linked to various pathological conditions, including osteoporosis, impaired wound healing, increased susceptibility to infections, and inflammatory skin diseases.^65^ Cellular senescence, a hallmark of aging, has been shown to interfere with VDR signaling through the accumulation of inflammatory mediators and oxidative stress, creating a feedback loop that further compromises vitamin D responsiveness.^66^ Additionally, age‐related changes in the gut microbiome can affect vitamin D metabolism and VDR function, as certain bacterial species are known to influence vitamin D absorption and VDR activity.^67^, ^68^ Understanding these age‐related alterations in the vitamin D/VDR system is crucial for developing targeted interventions to maintain optimal vitamin D signaling in elderly populations and prevent age‐associated diseases.

## 3 CELLULAR SENESCENCE AND INFLAMMATION IN AGING SKIN

### 3.1 Fundamental mechanisms of cellular senescence in skin aging

Molecular triggers: DNA damage and telomere shortening represent primary triggers of cellular senescence, characterized by the accumulation of DNA double‐strand breaks and progressive telomere erosion during cell division.^69^ This triggers the DNA damage response pathway, activating p53 and p21, which ultimately leads to cell cycle arrest.^70^ Oxidative stress and ROS accumulation significantly contribute to senescence through direct damage to cellular components and activation of stress response pathways.^71^ The generation of ROS exceeds cellular antioxidant defenses during aging, leading to oxidative damage of proteins, lipids, and DNA.^71^ Mitochondrial dysfunction and metabolic changes play crucial roles in senescence progression, with age‐related decline in mitochondrial function leading to reduced ATP production and increased ROS generation. These metabolic alterations trigger retrograde signaling that reinforces the senescent phenotype in age‐related diseases.^72^

Senescence‐associated secretory phenotype (SASP): The SASP represents a complex secretory program that fundamentally alters the tissue microenvironment through the release of various factors.^73^ Pro‐inflammatory cytokines and chemokines, including IL‐6, IL‐8, and TNF‐α, constitute major SASP components that promote inflammation and recruit immune cells.^74^ Matrix metalloproteinases (MMPs) secreted by senescent cells contribute to extracellular matrix degradation and tissue remodeling.^75^ Additional SASP factors include growth factors and other mediators such as VEGF, FGF, and PAI‐1, which influence neighboring cell behavior and tissue homeostasis.^76^ This complex secretome creates a pro‐inflammatory environment that can propagate senescence to surrounding cells through paracrine effects.

Impact on skin structure and function: Senescence significantly impacts skin structure through alterations in the extracellular matrix, including reduced collagen synthesis, increased matrix degradation, and altered cross‐linking of matrix proteins.^77^, ^78^ These changes result in decreased skin elasticity and strength. Changes in skin barrier function occur through compromised tight junction proteins, altered lipid composition, and reduced natural moisturizing factors.^79^ The impact on wound healing is particularly significant, with senescent cells impairing the normal wound repair process through delayed reepithelialization, reduced angiogenesis, and impaired matrix remodeling.^80^ The accumulation of senescent cells in aged skin contributes to chronic inflammation and delayed wound resolution.^81^

### 3.2 Inflammatory mechanisms and immunosenescence in skin aging

Age‐related immune changes: The aging process profoundly affects both innate and adaptive immune responses in the skin. Alterations in innate immunity include reduced neutrophil chemotaxis, impaired phagocytic activity, and decreased pattern recognition receptor function.^82^ Langerhans cells and dermal dendritic cells show reduced migration capacity and antigen‐presenting ability with age. Natural killer cell cytotoxicity and cytokine production also decline significantly.^83^ Changes in adaptive immune response manifest as decreased T cell repertoire diversity, reduced naive T cell production, and increased memory T cell accumulation. B cell responses become less efficient, with reduced antibody production and decreased specificity.^84^ These changes collectively impact skin immune surveillance, leading to increased susceptibility to infections, impaired tumor immunosurveillance, and delayed wound healing responses.^85^

**FIGURE 2:**
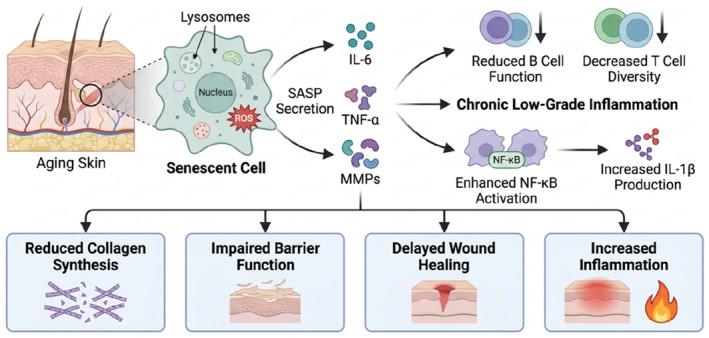
Cellular senescence and inflammation in aging skin. This schematic representation illustrates the complex interplay between cellular senescence and inflammatory processes in aging skin, where senescent cells secrete various senescence‐associated secretory phenotype factors including IL‐6, TNF‐α, and MMPs. These factors contribute to chronic low‐grade inflammation, characterized by decreased T cell diversity and reduced B cell function, alongside enhanced NF‐κB activation leading to increased IL‐1β production. The cumulative effects on dermal tissues, shown in the boxes below, include reduced collagen synthesis, impaired barrier function, delayed wound healing, and increased inflammation, demonstrating the profound impact of senescence‐driven inflammation on skin homeostasis during aging.

Inflammaging processes: Inflammaging is characterized by chronic low‐grade inflammation that develops with advancing age. This process is marked by persistent activation of inflammatory pathways, particularly the nuclear factor kappa B (NF‐κB) signaling cascade, which serves as a master regulator of inflammatory responses.^86^ The constitutive activation of NF‐κB leads to increased production of pro‐inflammatory mediators, including IL‐6, TNF‐α, and IL‐1β. Environmental factors, cellular senescence, and accumulated tissue damage contribute to this inflammatory state^87^ (Figure 2). The skin microbiome changes associated with aging can further exacerbate inflammatory processes.^88^

Impact on skin homeostasis: The combined effects of immunosenescence and inflammaging significantly disrupt skin homeostasis. These changes manifest as impaired barrier function, delayed wound healing, and increased susceptibility to skin infections and malignancies.^89^ The persistent inflammatory environment promotes extracellular matrix degradation through increased matrix metalloproteinase activity.^90^ Chronic inflammation also affects melanocyte function and epidermal stem cell maintenance, leading to pigmentary changes and reduced regenerative capacity.^91^ The dysregulation of immune responses contributes to the development of age‐related skin conditions such as xerosis, pruritus, and various inflammatory dermatoses.^92^

## 4 VDR‐DEPENDENT REGULATION OF INFLAMMATION AND SENESCENCE IN SKIN AGING AND DISEASES

### 4.1 VDR action in protecting against inflammation and senescence

The VDR exerts its protective effects against inflammation and senescence through multiple transcriptional mechanisms. Through direct target genes, VDR regulates the expression of key inflammatory mediators and senescence‐associated factors, with recent evidence showing its crucial role in DNA repair during oncogene‐induced senescence.^93^ The VDR/vitamin D axis acts as a protective shield against aging by modulating various cellular processes and inflammatory responses,^94^ and emerging research has begun to decode its complex role in targeting multiple hallmarks of aging.^66^

A novel VDR‐Ezh2‐p16 signaling axis has been reported to protect against age‐related osteoporosis and cellular senescence.^95^ Mechanistically, 1α,25(OH)_2_D_3_‐activated VDR binds directly to a vitamin D response element (VDRE) in the promoter region of the EZH2 gene, transcriptionally upregulating enhancer of zeste homolog 2 (Ezh2), the catalytic subunit of polycomb repressive complex 2 (PRC2). Ezh2 then catalyzes the trimethylation of histone H3 at lysine 27 (H3K27me3) at the CDKN2A locus, leading to epigenetic silencing of p16^INK4a^ expression. Because p16^INK4a^ is a key effector of irreversible cell cycle arrest and a hallmark of cellular senescence, this VDR‐Ezh2‐p16 axis functions as a critical anti‐senescence mechanism.^95^ While this pathway was initially characterized in bone‐derived cells, its relevance to skin aging is supported by the observation that age‐related decline in cutaneous VDR expression could similarly lead to Ezh2 downregulation and p16^INK4a^ accumulation in dermal fibroblasts and epidermal stem cells, contributing to skin senescence and impaired regenerative capacity. Recent findings also demonstrate VDR's capacity to constrain inflammation through modulation of key genes in specific chromosomal regions.^96^ The recruitment of co‐regulatory factors by VDR influences transcriptional responses during inflammation and cellular aging,^97^ with evidence showing that vitamin D3 can inhibit senescence‐associated inflammatory mediator secretion through specific pathway regulation.^98^ Epigenetic modifications orchestrated by VDR involve changes in DNA methylation patterns and histone modifications that influence age‐related gene expression programs, as demonstrated in studies of aging‐related osteoarthritis.^99^ Given the conserved transcriptional mechanisms by VDR, modulating this receptor system could potentially offer novel therapeutic strategies to combat skin aging by simultaneously addressing multiple aspects of senescence, inflammation, and epigenetic alterations in cutaneous tissues.

VDR signaling demonstrates extensive cross talk with major inflammatory and senescence‐related pathways. Chen et al.^100^ demonstrated that VDR physically interacts with IκB kinase *β* (IKKβ) through its ligand‐binding domain (specifically, helices H3–H5 of the VDR‐LBD), and this direct protein–protein interaction prevents IKKβ from phosphorylating IκBα, thereby blocking the nuclear translocation of NF‐κB p65/p50 heterodimers. This mechanism is ligand‐dependent, as 1α,25(OH)_2_D_3_ binding stabilizes VDR in a conformation that enhances its affinity for IKKβ.^100^ In senescent dermal fibroblasts, Sayegh et al.^98^ demonstrated that vitamin D_3_ acts through VDR to inhibit p38 MAPK phosphorylation at its Thr180/Tyr182 activation motif, thereby reducing secretion of SASP factors including IL‐6 and IL‐8. The underlying mechanism involves VDR transcriptionally upregulating MAPK phosphatase‐1 (MKP‐1/DUSP1), a dual‐specificity phosphatase that directly dephosphorylates p38 MAPK.^98^ VDR can also modulate upstream signaling by suppressing the activity of the direct kinase activators of p38. In dermal fibroblasts, VDR‐mediated p38 MAPK inhibition specifically reduces the transcription of MMP‐1, MMP‐3, and MMP‐9, thereby preserving extracellular matrix integrity in aging dermis.^98^

VDR signaling intersects with the PI3K/Akt pathway through both genomic and rapid non‐genomic mechanisms to regulate cell survival and prevent cellular senescence. In the non‐genomic pathway, 1α,25(OH)_2_D_3_ binds to caveolae‐associated VDR at the plasma membrane, which recruits and activates c‐Src kinase. c‐Src then activates PI3K by phosphorylating the p85 regulatory subunit (PIK3R1), leading to Akt phosphorylation at Ser473.^101^, ^102^ Activated Akt subsequently phosphorylates and inactivates the pro‐apoptotic factors FOXO3a and GSK‐3β, resulting in increased expression of antioxidant enzymes such as SOD2 and catalase, and suppression of pro‐apoptotic proteins including Bax and Bad.^101^, ^102^ Genomically, VDR can transcriptionally upregulate PIK3R1 (encoding the p85 subunit) and NFE2L2 (encoding Nrf2), thereby enhancing the cellular antioxidant defense system through downstream targets such as HO‐1 and NQO1. In keratinocytes, VDR‐PI3K/Akt signaling promotes cell survival after UV irradiation by upregulating Nrf2‐driven antioxidant genes and maintaining mTOR‐dependent autophagy. In epidermal stem cells, PI3K/Akt activation by VDR is essential for maintaining self‐renewal and preventing premature senescence, particularly through Akt‐mediated phosphorylation of p21, which promotes its cytoplasmic sequestration rather than nuclear accumulation, thereby avoiding cell cycle arrest.^101^, ^102^

A critical aspect of this network involves VDR's direct molecular interaction with p53 signaling. VDR and p53 physically interact through the VDR DBD and the p53 C‐terminal regulatory domain (amino acids 363–393), as demonstrated by co‐immunoprecipitation and GST pull‐down assays.^103^ This interaction has dual functional consequences. First, VDR can stabilize p53 under conditions of DNA damage by competing with MDM2 for p53 binding, thereby reducing p53 ubiquitination and proteasomal degradation. Second, VDR and p53 cooperatively bind to composite response elements in the promoters of DNA repair genes, including GADD45A (growth arrest and DNA damage‐inducible 45 alpha), thereby promoting nucleotide excision repair.^93^, ^103^ Graziano et al.^93^ showed that VDR is recruited to DNA damage sites during oncogene‐induced senescence, where it facilitates repair through upregulation of XPC and DDB2, two critical nucleotide excision repair genes. Key target genes co‐regulated by the VDR‐p53 axis include CDKN1A/p21 (cell cycle arrest), GADD45A (DNA repair), BAX (apoptosis), and BBC3/PUMA (apoptosis). Notably, VDR also limits excessive p53‐driven apoptosis by transcriptionally upregulating MDM2 expression in a context‐dependent manner, particularly after low‐level UV exposure.^103^ Collectively, this signaling network not only maintains skin barrier function but also protects against photoaging and carcinogenesis, suggesting that targeting the VDR‐p53 axis could offer therapeutic potential for age‐related skin conditions.

VDR's interaction with innate immunity pathways represents another crucial aspect of its antiaging effects, particularly through modulation of inflammatory mediators and immune cell function.^58^ Recent evidence has revealed important cross talk between VDR and p63 signaling pathways at the molecular level. Oda et al.^104^ demonstrated that VDR and the ΔNp63α isoform physically interact through their respective DNA‐binding domains and co‐occupy regulatory elements at epidermal differentiation gene loci. These composite VDRE/p63RE elements are found in the promoters of key differentiation genes including KRT1, KRT10, IVL (involucrin), and LOR (loricrin). VDR activation by 1α,25(OH)_2_D_3_ shifts ΔNp63α from its proliferative gene targets (KRT14 and KRT5, which mark basal undifferentiated keratinocytes) to differentiation gene targets, thereby promoting epidermal maturation and stratification.^104^ This mechanism is predominantly keratinocyte‐specific and represents a key molecular switch between proliferation and differentiation in the epidermis. In the context of skin aging, disruption of this VDR‐p63 axis may contribute to impaired epidermal differentiation and barrier dysfunction observed in aged skin. Furthermore, VDR exhibits reciprocal regulatory cross talk with the aryl hydrocarbon receptor (AHR) signaling pathway in keratinocytes. Christofi et al.^105^ demonstrated that AHR activation by environmental pollutants such as TCDD and benzo[a]pyrene transcriptionally downregulates VDR expression, while VDR activation by 1α,25(OH)_2_D_3_ suppresses AHR‐driven expression of xenobiotic‐metabolizing genes CYP1A1 and CYP1B1. This antagonism occurs at the level of shared co‐regulatory proteins: Both AHR (via its partner ARNT) and VDR (via RXR) compete for limiting amounts of the coactivators CBP/p300, creating a mutually inhibitory regulatory circuit.^105^ This mechanism is most relevant in keratinocytes and may extend to Langerhans cells. In aged skin, chronic AHR activation by accumulated environmental toxins may therefore suppress VDR activity, reducing VDR‐dependent anti‐inflammatory and pro‐differentiation programs while enhancing AHR‐driven pro‐inflammatory and pro‐oxidant gene expression, thereby creating a pro‐aging microenvironment.

**FIGURE 3:**
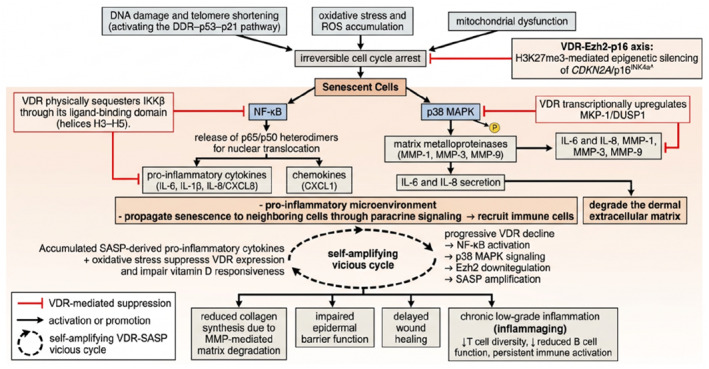
Bidirectional regulatory circuit between vitamin D receptor (VDR) signaling, cellular senescence, and inflammation in aging skin. This schematic illustrates the complex interplay between cellular senescence, the senescence‐associated secretory phenotype (SASP), and VDR‐mediated protective mechanisms in aging skin. In the upper portion, key molecular triggers of cellular senescence are depicted, including DNA damage and telomere shortening (activating the DDR–p53–p21 pathway), oxidative stress and ROS accumulation, and mitochondrial dysfunction, all of which converge on irreversible cell cycle arrest. Senescent cells produce SASP factors through two major transcriptional drivers: NF‐κB and p38 MAPK. Upstream activation of NF‐κB occurs through IKKβ‐mediated phosphorylation and degradation of IκBα, releasing p65/p50 heterodimers for nuclear translocation, which drives transcription of pro‐inflammatory cytokines (IL‐6, IL‐1β, and IL‐8/CXCL8) and chemokines (CXCL1). Simultaneously, p38 MAPK activation promotes transcription of matrix metalloproteinases (MMP‐1, MMP‐3, and MMP‐9) and further reinforces IL‐6 and IL‐8 secretion. These SASP factors collectively create a pro‐inflammatory microenvironment that propagates senescence to neighboring cells through paracrine signaling, recruits immune cells, and degrades the dermal extracellular matrix. VDR‐mediated protective mechanisms are shown counteracting this process at multiple nodes (indicated by inhibitory arrows): VDR physically sequesters IKKβ through its ligand‐binding domain (helices H3–H5), preventing NF‐κB nuclear translocation and suppressing transcription of IL‐6, IL‐1β, and IL‐8; VDR transcriptionally upregulates MKP‐1/DUSP1, which dephosphorylates and inactivates p38 MAPK, reducing secretion of IL‐6, IL‐8, MMP‐1, MMP‐3, and MMP‐9; and the VDR‐Ezh2‐p16 axis maintains H3K27me3‐mediated epigenetic silencing of CDKN2A/p16^INK4a^, preventing the entry into irreversible senescence and consequent SASP activation. The lower portion depicts the SASP‐to‐VDR feedback loop: Accumulated SASP‐derived pro‐inflammatory cytokines and oxidative stress suppress VDR expression and impair vitamin D responsiveness, creating a self‐amplifying vicious cycle (indicated by the circular arrow) in which progressive VDR decline leads to escalating NF‐κB activation, p38 MAPK signaling, enhancer of zeste homolog 2 downregulation, and SASP amplification. The downstream consequences for aged skin are shown in the boxes below, including reduced collagen synthesis due to MMP‐mediated matrix degradation, impaired epidermal barrier function, delayed wound healing, chronic low‐grade inflammation (inflammaging), and persistent immune activation.

Of note, these mechanisms may form a bidirectional regulatory circuit between VDR and the SASP. On the suppressive arm, VDR inhibits SASP through two converging routes: VDR–IKKβ physical interaction blocks NF‐κB nuclear translocation, suppressing transcription of IL‐6, IL‐1β, and IL‐8/CXCL8,^100^ while VDR‐mediated upregulation of MKP‐1/DUSP1 inactivates p38 MAPK, specifically reducing secretion of IL‐6, IL‐8, MMP‐1, MMP‐3, and MMP‐9 from senescent fibroblasts.^98^ MMP suppression is particularly relevant in the skin, as these proteases drive dermal collagen degradation and can proteolytically activate latent SASP factors, further amplifying inflammation.^75^ Upstream of SASP production itself, the VDR‐Ezh2‐p16 axis prevents p16^INK4a^‐driven irreversible senescence and consequent SASP activation, as confirmed by multiomics profiling showing that VDR restoration re‐establishes Ezh2 expression and attenuates IL‐6, IL‐1β, and MMP‐3 secretion simultaneously.^95^, ^106^ On the feedback arm, the chronic inflammatory milieu generated by SASP—particularly accumulated pro‐inflammatory cytokines and oxidative stress—further suppresses VDR expression and impairs vitamin D responsiveness,^66^ creating a self‐amplifying vicious cycle in which progressive VDR loss drives escalating NF‐κB activation, p38 signaling, Ezh2 downregulation, and SASP amplification, ultimately accelerating irreversible senescence in cutaneous cells (Figure 3). These intricate signaling networks collectively demonstrate that VDR functions as a central integrator of cellular stress responses and aging‐related signaling cascades, with specific molecular interactions, distinct target gene profiles, and cell‐type dependent outcomes in the skin. The detailed molecular understanding of these VDR pathway interactions provides a foundation for developing targeted therapeutic strategies that address specific aspects of inflammatory senescence‐associated skin aging.

### 4.2 Cell‐type specific regulation by VDR

**FIGURE 4:**
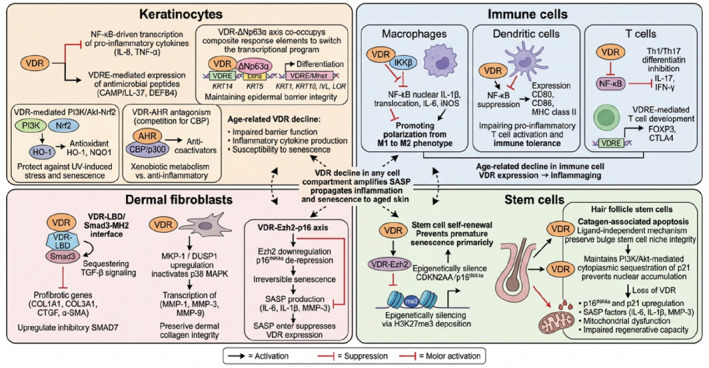
Cell‐type specific vitamin D receptor (VDR) regulation of inflammation and senescence in skin. This schematic illustrates the distinct yet interconnected roles of VDR signaling in four major skin cell populations, with emphasis on how VDR modulates inflammation and cellular senescence in each cell type through specific molecular targets and pathways. Keratinocytes (upper left): VDR activation suppresses NF‐κB‐driven transcription of pro‐inflammatory cytokines (IL‐8, TNF‐α) while simultaneously promoting VDRE‐mediated expression of antimicrobial peptides (CAMP/LL‐37 and DEFB4). The VDR‐ΔNp63α axis co‐occupies composite response elements to switch the transcriptional program from proliferation‐associated genes (KRT14 and KRT5) to differentiation‐associated genes (KRT1, KRT10, IVL, and LOR), maintaining epidermal barrier integrity. VDR‐mediated PI3K/Akt‐Nrf2 signaling upregulates antioxidant genes (HO‐1 and NQO1), protecting against UV‐induced oxidative stress and senescence. VDR‐AHR antagonism, through competition for CREB‐binding protein/p300 coactivators, balances xenobiotic metabolism against anti‐inflammatory gene expression. Age‐related VDR decline in keratinocytes leads to impaired barrier function, increased inflammatory cytokine production, and susceptibility to senescence. Immune cells (upper right): In macrophages, VDR‐IKKβ interaction blocks NF‐κB nuclear translocation, suppressing IL‐1β, IL‐6, and iNOS transcription and promoting polarization from pro‐inflammatory M1 to anti‐inflammatory M2 phenotype. In dendritic cells, VDR‐mediated NF‐κB suppression reduces expression of maturation markers (CD80, CD86, and MHC class II), impairing pro‐inflammatory T cell activation and promoting immune tolerance. In T cells, VDR suppresses Th1/Th17 differentiation through NF‐κB‐dependent inhibition of IL‐17 and IFN‐γ while promoting regulatory T cell development via VDRE‐mediated upregulation of FOXP3 and CTLA4. Age‐related decline in immune cell VDR expression contributes to inflammaging through loss of these immunoregulatory mechanisms. Dermal fibroblasts (lower left): VDR directly interacts with SMAD3 via the VDR‐LBD/SMAD3–MH2 interface, sequestering SMAD3 from TGF‐β signaling and suppressing profibrotic genes (COL1A1, COL3A1, CTGF, and *α*‐SMA), while transcriptionally upregulating the inhibitory SMAD7. VDR‐mediated MKP‐1/DUSP1 upregulation inactivates p38 MAPK, reducing transcription of MMP‐1, MMP‐3, and MMP‐9 and thereby preserving dermal collagen integrity. The VDR‐Ezh2‐p16 axis is relevant in fibroblasts, where age‐related VDR decline leads to enhancer of zeste Homolog 2 downregulation, p16^INK4a^ derepression, irreversible senescence, and senescence‐associated secretory phenotype (SASP) production (IL‐6, IL‐1β, and MMP‐3), which further suppresses VDR expression, establishing the vicious cycle. Stem cells (lower right): VDR maintains stem cell self‐renewal and prevents premature senescence primarily through the VDR‐Ezh2‐p16 axis, which epigenetically silences CDKN2A/p16^INK4a^ via H3K27me3 deposition. In hair follicle stem cells, VDR controls catagen‐associated apoptosis through a ligand‐independent mechanism essential for preserving bulge stem cell niche integrity. VDR also maintains PI3K/Akt‐mediated cytoplasmic sequestration of p21, preventing its nuclear accumulation and cell cycle arrest. Loss of VDR in stem cells leads to p16^INK4a^ and p21 upregulation, elevated SASP factors (IL‐6, IL‐1β, and MMP‐3), mitochondrial dysfunction, and impaired regenerative capacity. The central connecting arrows illustrate that VDR decline in any cell compartment amplifies SASP production, which propagates inflammation and senescence to neighboring cell types through paracrine signaling, collectively driving the inflammatory senescence phenotype of aged skin.

Keratinocytes: Keratinocytes are the predominant cell type in the epidermis, forming multiple layers of the stratified epithelium and serving as the primary barrier between the body and the external environment. VDR signaling in keratinocytes plays a fundamental role in maintaining epidermal homeostasis, with both ligand‐dependent and ligand‐independent actions being essential for normal keratinocyte function^107^ (Figure 4). Studies using VDR knockout mice have demonstrated critical defects in keratinocyte proliferation and differentiation, highlighting an essential role of VDR signaling in maintaining proper epidermal turnover.^108^ Transcriptional profiling has revealed that VDR regulates a complex epidermal differentiation network, controlling the expression of numerous genes involved in barrier function and terminal differentiation.^109^ The receptor is particularly crucial for keratinocyte stem cell function, with VDR deficiency leading to impaired self‐renewal capacity and altered differentiation patterns.^110^ Recent research has shown that VDR deficiency in keratinocytes can augment dermal fibrosis and inflammation, suggesting a broader role for VDR in maintaining skin homeostasis and preventing pathological conditions.^111^ VDR activation in keratinocytes modulates the production of inflammatory mediators and antimicrobial peptides, thereby contributing to the skin's innate immune defense and maintaining proper inflammatory responses.^112^ At the molecular pathway level, keratinocyte‐specific VDR actions involve several distinct signaling interactions. VDR suppresses NF‐κB‐driven transcription of pro‐inflammatory cytokines (IL‐8 and TNF‐α) while simultaneously promoting expression of antimicrobial peptides (CAMP/LL‐37 and DEFB4) through direct VDRE‐mediated transcription.^100^, ^112^ The VDR‐p63 axis operates primarily in keratinocytes, where VDR‐ΔNp63α co‐occupancy of composite response elements switches the transcriptional program from proliferation‐associated genes (KRT14 and KRT5) to differentiation‐associated genes (KRT1, KRT10, IVL, and LOR).^104^ VDR‐AHR antagonism in keratinocytes modulates the balance between xenobiotic metabolism and anti‐inflammatory gene expression through competition for CBP/p300 coactivators.^105^ Additionally, VDR‐mediated activation of PI3K/Akt‐Nrf2 signaling in keratinocytes upregulates antioxidant genes (HO‐1 and NQO1) that provide protection against UV‐induced oxidative damage.^101^, ^102^ These findings suggest a potential role of VDR as a multi‐pathway integrator in keratinocyte homeostasis during aging.

Immune cells: VDR expression in immune cells orchestrates various aspects of both innate and adaptive immunity in the skin. In T cells, VDR signaling regulates differentiation pathways, promoting the development of regulatory T cells while suppressing pro‐inflammatory Th1 and Th17 responses.^48^ Dendritic cell function is significantly modulated by VDR activation, affecting their maturation, migration, and antigen‐presenting capabilities.^113^ The role of VDR in macrophages is particularly crucial, as demonstrated by evidence showing its essential function in regulating tissue‐resident macrophage responses to injury, which is vital for proper wound healing and tissue homeostasis .^114^ VDR signaling influences macrophage polarization, promoting an anti‐inflammatory M2 phenotype while suppressing pro‐inflammatory M1 activation.^115^ Recent evidence also highlights the importance of VDR in maintaining immunocompetence during aging,^116^ with its activity being crucial for preventing age‐related immune dysfunction and controlling inflammaging processes in skin tissue. At the molecular level, VDR‐mediated immunoregulation operates through cell‐type specific pathway interactions. In macrophages, VDR physically binds IKKβ through its ligand‐binding domain to block NF‐κB nuclear translocation, thereby suppressing transcription of IL‐1β, IL‐6, and iNOS, which contributes to the polarization shift from pro‐inflammatory M1 to the anti‐inflammatory M2 phenotype.^100^, ^115^ In dendritic cells, VDR‐mediated NF‐κB suppression reduces the expression of maturation markers (CD80, CD86, and MHC class II), impairing their capacity to activate pro‐inflammatory T cell responses and thereby promoting immune tolerance.^113^ In T cells, VDR activation suppresses Th1 and Th17 differentiation while promoting regulatory T cell development, partly through inhibition of NF‐κB‐dependent IL‐17 and IFN‐γ transcription and partly through direct VDRE‐mediated upregulation of FOXP3 and CTLA4.^48^ These immune cell‐specific VDR pathway interactions may explain how vitamin D/VDR signaling maintains immune homeostasis in the skin and how age‐related decline in VDR expression contributes to inflammaging.

Dermal fibroblasts: Dermal fibroblasts are key mesenchymal cells residing in the dermis that play essential roles in maintaining skin structure, producing extracellular matrix components, and orchestrating wound healing responses. In these cells, VDR signaling orchestrates complex regulatory networks that control both physiological and pathological processes. A recent study revealed that novel vitamin D3 hydroxymetabolites exert their antifibrogenic activities through VDR‐dependent mechanisms, highlighting the crucial role of VDR in preventing excessive fibrosis of fibroblasts.^117^ VDR signaling demonstrates important cross talk with TGF‐β1 pathways, where it modulates SMAD signaling and associated fibrogenic responses.^118^ The interaction between vitamin D and TGF‐β1 has been shown to synergistically promote dermal wound healing, with evidence suggesting a significant modulatory effect on fibroblast function.^119^, ^120^ VDR directly interacts with SMAD3 via the VDR ligand‐binding domain and the SMAD3 MH2 domain, sequestering SMAD3 from the TGF‐β/Smad signaling complex and preventing its nuclear translocation and transcriptional activity on profibrotic target genes such as COL1A1, COL3A1, CTGF, and *α*‐SMA (ACTA2).^118^, ^121^ Additionally, VDR transcriptionally upregulates SMAD7, an inhibitory SMAD that blocks TGF‐β receptor type I‐mediated phosphorylation of SMAD2/3, thereby providing a second mechanism for attenuating profibrotic signaling.^121^ Beyond TGF‐β cross talk, VDR‐mediated inhibition of p38 MAPK signaling in dermal fibroblasts specifically reduces the transcription of MMP‐1, MMP‐3, and MMP‐9, thereby preserving extracellular matrix integrity in aging dermis.^98^ The VDR‐Ezh2‐p16 axis may also be relevant in dermal fibroblasts, as age‐related decline in VDR expression could lead to Ezh2 downregulation and accumulation of p16^INK4a^, contributing to fibroblast senescence and SASP production.^95^

Stem cells: VDR expression and signaling are essential regulators of various stem cell populations in the skin and beyond (Figure 4). During cutaneous wound repair, VDR plays a critical role in controlling epidermal stem cell proliferation, migration, and differentiation, processes that are fundamental for proper tissue regeneration.^122^ VDR is essential for maintaining stem cell homeostasis through multiple mechanisms. Yang et al.^95^ identified a VDR‐Ezh2‐p16 epigenetic signaling axis in which 1α,25(OH)_2_D_3_‐activated VDR binds to a VDRE in the EZH2 promoter, upregulating Ezh2 (the catalytic subunit of PRC2), which deposits H3K27me3 at the CDKN2A locus to epigenetically silence p16^INK4a^ and prevent irreversible cell cycle arrest; loss of VDR disrupts this axis, leading to p16^INK4a^ derepression, premature senescence, and elevated SASP factor production.^95^ In hair follicle biology, Joko et al.^123^ demonstrated using VDR‐null mice that VDR controls the spatiotemporal activation of programmed cell death during catagen through a ligand‐independent mechanism. Its absence causes disrupted apoptosis, aberrant lower follicle retention, dermal cyst formation, and permanent alopecia, highlighting VDR's indispensable role in preserving bulge stem cell niche integrity beyond its functions in anagen initiation.^123^ Multiomics profiling by Chen et al.^106^ further confirmed VDR as a central senescence regulatory hub, showing that VDR downregulation leads to upregulation of p16^INK4a^ and p21, elevated SASP factors (IL‐6, IL‐1β, and MMP‐3), and mitochondrial dysfunction, while VDR restoration partially rescued these phenotypes.^106^ Collectively, these findings position VDR as a master regulator connecting epigenetic maintenance, cell cycle control, inflammatory signaling, and metabolic homeostasis in stem cells, with direct implications for cutaneous stem cell populations where age‐related VDR decline may drive senescence and impaired skin regeneration.

### 4.3 Disease‐specific actions of VDR

Inflammatory conditions: VDR signaling plays crucial roles in various inflammatory skin conditions through distinct molecular mechanisms. In psoriasis, VDR activation regulates keratinocyte proliferation and differentiation while suppressing the IL‐23/IL‐17 axis and modulating T cell responses.^124^ In atopic dermatitis, VDR activation has been shown to improve allergen‐triggered eczematous inflammation through modulation of T cell responses and inflammatory mediators, as demonstrated in experimental models.^125^ Research has revealed that the immunomodulatory effects of VDR in inflammatory skin conditions operate through multiple mechanisms: regulation of antimicrobial peptide expression, modulation of T cell differentiation, inhibition of dendritic cell maturation, and suppression of pro‐inflammatory cytokine production.^126^ These mechanisms are particularly relevant in contact dermatitis and other inflammatory skin conditions, where VDR signaling helps maintain immune homeostasis and skin barrier function. The therapeutic implications of VDR pathway modulation in inflammatory skin diseases are also supported by its ability to promote an anti‐inflammatory environment through enhancement of regulatory T cell function and suppression of excessive immune responses.^126^

Age‐related conditions: The involvement of VDR signaling in age‐related skin conditions encompasses multiple pathophysiological processes. In photoaging, VDR signaling could protect against UV‐induced DNA damage and oxidative stress.^127^, ^128^ VDR signaling has also been shown to ameliorate cellular phenotypes associated with accelerated aging, as demonstrated in Hutchinson–Gilford progeria syndrome, where it improves nuclear architecture and reduces DNA damage accumulation.^129^ The role of VDR in chronic wound healing involves complex regulation of multiple cellular processes. VDR expression is essential for proper epidermal stem cell function during wound repair, including their proliferation, migration, and differentiation, which are crucial for efficient reepithelialization.^122^ Furthermore, VDR signaling plays a critical role in modulating the angiogenic response during wound healing by regulating endothelial cell function and limiting excessive inflammatory responses.^130^ Studies have demonstrated that both vitamin D deficiency and VDR knockout significantly delay wound healing through impaired epithelial repair and reduced nerve density, highlighting the essential role of vitamin D/VDR signaling in tissue repair.^131^, ^132^ Notably, in skin carcinogenesis, VDR demonstrates tumor‐suppressive functions through regulation of cell cycle arrest, apoptosis, and DNA repair mechanisms.^133^ This is supported by the observation that VDR knockout mice demonstrated enhanced susceptibility to UV‐induced skin tumorigenesis and inflammation.^134^, ^135^ Additionally, VDR signaling has been shown to inhibit the epithelial–mesenchymal transition.^136^ Collectively, these findings underscore the critical importance of maintaining adequate vitamin D/VDR signaling in aged skin for optimal tissue repair, photoprotection, and cancer prevention, suggesting potential therapeutic applications in age‐related skin conditions.

## 5 THERAPEUTIC APPLICATIONS OF MODULATING VDR SIGNALING

### 5.1 Current vitamin D‐based treatments

The therapeutic applications of vitamin D and its analogs in skin conditions have evolved significantly over recent decades. Topical vitamin D analogs, particularly calcipotriol and calcitriol, remain first‐line treatments for psoriasis, either as monotherapy or in combination with corticosteroids.^137^ These compounds exert their therapeutic effects through modulation of keratinocyte proliferation and differentiation, as well as immunomodulatory actions on T cells and dendritic cells.^138^ The combination of calcipotriol with betamethasone dipropionate has shown superior efficacy to either agent alone in treating plaque psoriasis, leading to the development of various formulations including ointments, gels, and foams for enhanced patient compliance.^139^ In photoaging and skin cancer prevention, topical vitamin D compounds have demonstrated promising results in reducing UV‐induced DNA damage and inflammation, although their clinical application in this context remains under investigation.^140^ For chronic wounds, both topical and systemic vitamin D supplementation strategies have been explored, with some studies showing improved healing outcomes in diabetic wounds and pressure ulcers.^141^, ^142^ Novel delivery systems, such as nanoformulations and vitamin D analog–containing dressings, are being developed to enhance the therapeutic efficacy and tissue penetration of these compounds.^143^ However, the use of vitamin D–based treatments requires careful monitoring of calcium homeostasis, particularly with systemic administration, and consideration of potential adverse effects such as skin irritation with topical applications.^144^

### 5.2 Modulation of the VDR signaling by bioactive compounds

Bioactive compounds derived from natural products or traditional Chinese medicine have shown promising effects in modulating VDR signaling in skin conditions and malignancy.^18^ In the context of inflammatory skin conditions like psoriasis, several TCM compounds and formulations have demonstrated therapeutic potential through VDR‐dependent pathways. For instance, berberine has been found to regulate keratinocyte inflammation through modulation of the p38 MAPK/NF‐κB pathway, which intersects with VDR signaling.^145^ Traditional Chinese medicine formulations such as Jueyin granules have shown effectiveness in treating psoriasis by upregulating VDR expression and downregulating inflammatory pathways.^146^ These findings align with comprehensive reviews of TCM treatments for psoriasis, which highlight the importance of targeting multiple pathways, including VDR‐mediated signaling, for effective treatment outcomes.^147^ In skin cancer prevention, bufalin has demonstrated remarkable effects by stabilizing nuclear VDR expression and enhancing VDR‐mediated tumor suppression.^140^ The significance of VDR modulation in skin cancer treatment is further underscored by recent findings showing that VDR knockdown enhances melanoma malignancy and reduces responsiveness to vitamin D derivatives, highlighting the critical role of VDR‐targeting compounds in cancer therapy.^148^

The mechanisms by which these compounds modulate VDR signaling are diverse and extend beyond skin conditions. For example, asperuloside has been shown to enhance VDR expression in the colon and inhibit epithelial–mesenchymal transition via VDR/SMAD3 pathway regulation, demonstrating potential in treating inflammatory bowel conditions and colorectal cancer.^149^ Similarly, Astragalus polysaccharide upregulates adrenocortical VDR activity and vitamin D levels, which suppresses inflammatory cytokines and oxidative stress in sepsis models.^150^ Based on the attached study, in diabetic nephropathy, parthenolide regulates VDR expression through DNMT1‐mediated methylation, which helps improve podocyte function and reduce renal damage.^151^ Given these diverse mechanisms of VDR modulation by natural compounds, it is plausible that they could also be effective in treating inflammatory senescence‐associated skin aging by targeting VDR‐dependent pathways. The ability of these compounds to regulate both inflammatory responses and epigenetic modifications through VDR signaling suggests potential therapeutic applications in age‐related skin conditions where chronic inflammation and altered VDR signaling play key roles. Notably, while these compounds demonstrate diverse VDR‐modulatory activities through distinct pathways, most studies have not yet dissected the precise molecular mechanisms at the level of coactivator/corepressor recruitment or chromatin remodeling. Future investigations that systematically characterize and compare these mechanistic details will be essential for optimizing the therapeutic application of TCM‐derived compounds in inflammatory senescence‐associated skin aging.

### 5.3 Novel therapeutic strategies targeting VDR

Research on VDR‐targeted therapies has revealed multiple therapeutic mechanisms, with recent discoveries showing that compounds such as EB1089 can function independently of VDR pathways,^152^ while structural modifications of vitamin D analogs demonstrate enhanced interactions with variant VDR forms.^153^ These findings have been validated through sophisticated screening platforms.^154^, ^155^ Although these VDR ligands show promise in treating various conditions including cancer and inflammatory diseases with tissue‐specific effects,^156^ their potential applications in inflammatory senescence‐associated skin aging require further exploration.

Innovation in drug delivery systems has significantly advanced vitamin D–based therapeutics.^157^ Enhanced follicular delivery has been achieved through liposomal formulations and novel penetration enhancers.^158^ Computer‐aided screening of permeation enhancers using molecular dynamics has improved predictions of skin penetration.^159^ Transdermal vitamin D3 formulation studies have focused on optimizing skin retention and permeation profiles.^160^ Niosomal systems offer advantages for vitamin D delivery due to their nonionic properties,^161^ while nanocrystal formulations enhance bioavailability of poorly soluble analogs.^162^ Furthermore, gene therapy approaches including keratinocyte‐targeted VDR expression^163^ and VDR‐expressing viral vectors^164^ show therapeutic potential in experimental models. The integration of advanced delivery systems with these targeted approaches may provide novel treatment strategies for cellular senescence and inflammation in aging skin.

## 6 FUTURE PERSPECTIVES AND CONCLUSIONS

Despite significant advances in understanding role of VDR in inflammatory senescence‐associated skin aging, several critical knowledge gaps remain. The complex interplay between VDR and age‐related changes in inflammatory signaling pathways requires further investigation, particularly regarding the temporal and spatial specificity of VDR actions during different stages of skin aging. Understanding how VDR polymorphisms influence individual responses to age‐related skin inflammation and treatment outcomes is crucial for developing personalized therapeutic approaches. Additionally, the relationship between systemic vitamin D levels and local VDR signaling in aged skin remains poorly defined, especially concerning how circulating vitamin D metabolites influence VDR‐mediated transcriptional activity in different skin cell populations during the aging process.

Current technical limitations pose significant challenges in advancing VDR research in skin aging. Real‐time monitoring of VDR activity in aging skin remains difficult due to limitations in imaging techniques. While various animal models and in vitro systems exist, they often fail to accurately represent the complexity of human skin aging and associated inflammatory processes. The development of tissue‐specific delivery systems for aged skin presents another major challenge, as creating formulations that effectively target senescent cells while minimizing systemic exposure remains difficult. The complex architecture of aged skin, variations in penetration, and maintaining therapeutic concentrations at target sites all contribute to these challenges.

Future research priorities should focus on developing improved methods for studying VDR signaling in aging human skin, particularly through single‐cell technologies to understand cell‐specific responses. Emphasis should be placed on exploring combination therapies that target multiple pathways alongside VDR signaling, potentially leading to more effective treatments for inflammatory senescence‐associated skin aging. The development of personalized treatment strategies based on VDR genetic polymorphisms and aging‐related biomarkers will be crucial for optimizing therapeutic outcomes. As research continues to unravel the complexities of VDR signaling in skin aging, the integration of molecular insights with clinical applications will be essential for developing more effective, personalized treatments for age‐related skin conditions.

## AUTHOR CONTRIBUTIONS

The authors confirm contribution to the paper as follows: study conception and design: Fan Yang and Meijuan Li; draft manuscript preparation: Liancheng Guan and Fan Yang; Literature collation: Yujia Chen, Deping Luo and Hongxia Li; Framework: Zexin Zhao and Qian Li; review and editing manuscript: Yunzhi Chen. All authors reviewed the results and approved the final version of the manuscript.

## CONFLICT OF INTEREST STATEMENT

The authors declare no conflicts of interest.

## ETHICS STATEMENT

Not applicable.

## Data Availability

The data that support the findings of this study are available from the corresponding author upon reasonable request.
